# Research training needs of psychiatry postgraduates in India: insights from a cross-sectional survey

**DOI:** 10.1192/j.eurpsy.2026.10965

**Published:** 2026-07-31

**Authors:** R. K. Gupta, J. Yadav, R. Gupta

**Affiliations:** 1 Institute Of Mental Health, Pt BD Sharma University of Health Sciences, Rohtak; 2 Psychiatry, Medical College, Bhiwani, India

## Abstract

**Introduction:**

There is hardly any exposure to research activities in undergraduate medical courses and dissertation writing lays foundation of research training in postgraduate academic career. In the postgraduate medical education in India, thesis projects are critical components of research activities.Therefore, it is crucial to assess the needs of young researchers to enhance the quality of research and provide conducive environment for their research ideas,

**Objectives:**

To explore the research training needs of Psychiatry postgraduate trainees; to understand their perceptions, experiences, and challenges shaping their research behaviour.

**Methods:**

A cross-sectional survey was conducted among Psychiatry postgraduate trainees across different states of India. Stratified random sampling with multistage approach was used for data collection. A validated survey questionnaire comprising of 32 items developed to address potential gaps in postgraduate research training ; having four domains: demographics, exposure to research principles, barriers faced during dissertation writing and an open ended question inviting suggestions for promoting good research practices. The responses were exported to Microsoft Excel and analysed using descriptive statistics. Categorical variables were presented as percentages, while mean and standard deviation were calculated for quantitative variables

**Results:**

Hundred Psychiatry postgraduate trainees completed the survey questionnaire. The mean age of respondents was 25.5±1.5 years. The majority of trainees reported ‘low exposure’ (n= 65, 65 %) to research methodology, with only 7% reporting ‘good exposure’ to research methodology before starting their dissertation writing. Furthermore, just 1% post graduate trainees were involved in the process of publication during under-graduation. The majority of survey respondents believed that the fundamental purpose of thesis writing is to expose postgraduate students to ‘research methodology’ (65%). 30% participants selected the topic based on their own spontaneous idea and rest of the trainees undertook topics suggested by their mentors. The most common factor which influenced their decision for finalising the topic of thesis was ‘feasibility’ (60%), outweighing novelty.

**Conclusions:**

The study findings reflect a complex interplay of limited early exposure to research methodology, mentor-driven research culture and a growing dependence on digital tools. While most students recognised that thesis writing is intended to develop research skills, many approached it as an obligatory formality. With AI playing a major role in contemporary research, originality and novelty of research is compromised. It is imperative to strengthen structured research training, encourage independent ideas and foster supportive mentorship to cultivate originality and develop scientific temper among postgraduate psychiatry residents.

**Disclosure of Interest:**

R. K. Gupta Consultant of: none, J. Yadav Employee of: none, R. Gupta Employee of: none

